# Bioinformatics Analysis Reveals Crosstalk Among Platelets, Immune Cells, and the Glomerulus That May Play an Important Role in the Development of Diabetic Nephropathy

**DOI:** 10.3389/fmed.2021.657918

**Published:** 2021-06-24

**Authors:** Xinyue Yao, Hong Shen, Fukai Cao, Hailan He, Boyu Li, Haojun Zhang, Xinduo Zhang, Zhiguo Li

**Affiliations:** ^1^The Hebei Key Lab for Organ Fibrosis, The Hebei Key Lab for Chronic Disease, School of Public Health, International Science and Technology Cooperation Base of Geriatric Medicine, North China University of Science and Technology, Tangshan, China; ^2^Department of Modern Technology and Education Center, North China University of Science and Technology, Tangshan, China; ^3^Department of Jitang College, North China University of Science and Technology, Tangshan, China; ^4^Beijing Key Lab for Immune-Mediated Inflammatory Diseases, Institute of Clinical Medical Sciences, China-Japan Friendship Hospital, Beijing, China

**Keywords:** diabetic nephropathy, pathogenesis, bioinformatics, platelet, glomerulus, immune cell

## Abstract

Diabetic nephropathy (DN) is the main cause of end stage renal disease (ESRD). Glomerulus damage is one of the primary pathological changes in DN. To reveal the gene expression alteration in the glomerulus involved in DN development, we screened the Gene Expression Omnibus (GEO) database up to December 2020. Eleven gene expression datasets about gene expression of the human DN glomerulus and its control were downloaded for further bioinformatics analysis. By using R language, all expression data were extracted and were further cross-platform normalized by Shambhala. Differentially expressed genes (DEGs) were identified by Student's *t*-test coupled with false discovery rate (FDR) (*P* < 0.05) and fold change (FC) ≥1.5. DEGs were further analyzed by the Database for Annotation, Visualization, and Integrated Discovery (DAVID) to enrich the Gene Ontology (GO) terms and Kyoto Encyclopedia of Genes and Genomes (KEGG) pathway. We further constructed a protein-protein interaction (PPI) network of DEGs to identify the core genes. We used digital cytometry software CIBERSORTx to analyze the infiltration of immune cells in DN. A total of 578 genes were identified as DEGs in this study. Thirteen were identified as core genes, in which *LYZ, LUM*, and *THBS2* were seldom linked with DN. Based on the result of GO, KEGG enrichment, and CIBERSORTx immune cells infiltration analysis, we hypothesize that positive feedback may form among the glomerulus, platelets, and immune cells. This vicious cycle may damage the glomerulus persistently even after the initial high glucose damage was removed. Studying the genes and pathway reported in this study may shed light on new knowledge of DN pathogenesis.

## Introduction

Diabetic nephropathy (DN) is one of the most serious diabetic chronic microvascular complications and the major cause of end stage renal disease (ESRD) (1, 2). Glomerulus damage is one of the primary pathological changes in DN (3). The progression of DN is known to occur in a series of pathological changes in the glomerulus, such as expansion of glomerular mesangium, glomerular basement membrane (GBM) thickness, and podocytes loss. These changes damage glomerular filtration, causing proteinuria and glomerulosclerosis. Eventually, this may cause a decrease in the glomerular filtration rate (GFR) and the development of end stage renal disease (4). Now, DN is the main reason for dialysis or a kidney transplant and is a great global public health burden (5). Currently, drugs only target the renin-angiotensin-aldosterone system (RAAS) and sodium-glucose cotransporter 2 (SGLT2) inhibitors to treat DN (5–7). Therefore, it is urgent to explore the newly found molecular mechanism of DN and provide a new target for the diagnosis and treatment of DN.

Transcriptomic analysis is a powerful tool used to discover new targets and explore many diseases including DN (8). A lot of work has been done using the transcriptomic method, which has provided some novel targets and mechanisms for DN (9–17). However, as is known, the transcriptomic method has some limitations. This method can only use a single race sample and a small sample number, which is disproportionate to their high costs. And most transcriptomic methods have poor instability for their great measurement error (9). So, gene screening by different transcriptomic research methods vary and even conflict sometimes. Bioinformatics tools can integrate multiple transcriptomic methods to increase the statistical power. So, reduced population samples and many stable differentially expressed genes can be obtained (17). Bioinformatic tools were used in a lot of studies to analyze existing transcriptomics data and some important discoveries were found. Tang et al. analyzed glomerulus and renal tubule tissue transcription omics data, and found that NTNG1 and HGF were potential DN biomarkers of high specificity and sensitivity (18). Wang et al. found that the glomerulus in DN kidney tissue mainly caused changes in cell connectivity and tissue cell modification, while renal tubular tissue mainly caused abnormalities in energy metabolism, and changes in methylation status of core regulatory genes might be a potential factor for the pathogenesis of DN (19). As far as we know, research has seldom used bioinformatic tools to analyze all human existing glomerulus transcriptomics datasets to discover new potential biomarkers and the pathogenesis of DN. So it will be very attractive to do it.

Bioinformatic tools combining the information of multiple independent transcriptomic studies fundamentally include meta-analysis and cross-platform normalization (20). In the meta-analysis approach, each experiment is first analyzed separately and then combined by one of three types of statistics: *p*-value, effect size, and ranked gene lists. Cross-platform normalization considers all platform transcriptomic data as a single dataset. This approach normalizes transcriptomic data to remove the artifactual differences between transcriptomic studies and preserves biological differences between conditions. Cross-platform normalization is thought to have better performance than meta-analysis for “separate statistics” and “averaging is often less powerful than directly computing statistics from aggregated data” (20). We can always significantly find more differentially expressed genes in this process than meta-analysis (20). There is more than a dozen methods that can be used to undertake cross-platform normalization. But most of these cross-platform normalization methods can only process two different platforms, and transcriptomic data have comparable sample sizes. Recently a new method Shambhala (https://github.com/oncobox-admin/harmony) was found to solve this problem and may be the best choice to process gigantic transcriptomic datasets (21). Shambhala performs cross-platform normalization by using an auxiliary calibration dataset (P0) and a reference definitive dataset (Q). The initial data can be output into a generic form of a gene expression profile. This method can make experimental data independent of the experimental platform and the number of experiments (21). It can improve data comparability and reduce batch effect greatly (21). Above all, it is currently the only platform-independent data coordination technology that supports the processing of data obtained from multiple experimental platforms (21). Application of this technique may be the best choice to analysis all human existing glomerulus transcriptomics datasets.

Our objective is to comprehensively analyze transcriptomic profiles of all existing DN patient glomerular datasets in the GEO database for understanding of the pathogenesis of DN. In this study, we firstly downloaded all DN patients and their control glomerular original transcriptomic data. Then we used Bioconductor packages to extract the transcriptomic profiles and perform cross-platform normalization (Shambhala method), a static tests screening, and identification of differentially expressed genes (DEGs). We used the Database for Annotation, Visualization, and Integrated Discovery (DAVID) to enrich the Gene Ontology (GO) DEGs and the Kyoto Encyclopedia of Genes Genomes (KEGG) pathway. We constructed a protein-protein interaction (PPI) network and modules to screen core genes. We further used CIBERSORTx to explore the infiltration of immune cells in the DN glomerulus.

## Materials and Methods

### Dataset Selection

The Gene Expression Omnibus (GEO, http://www.ncbi.nlm.nih.gov/geo/) is an international public repository containing high-throughput microarray and next-generation sequence functional genomic datasets, which can provide researchers with a large number of gene expression profile data (22, 23). At present, a large number of microarray data of different diseases have been collected in the National Center for Biotechnology Information (NCBI, http://www.ncbi.nlm.nih.gov/) database for sharing and learning by institutes around the world. In this report, in order to obtain transcripts related to human DN, we searched all GEO datasets in the NCBI database with details of “diabetic nephropathy [Mesh],” “glomerular [Mesh],” and “Homo sapiens [porgn:__txid9606]” before December 2020. A total of 21 datasets were retrieved. We defined the following exclusion criteria: (i) cell line sample; (ii) a sample of biological fluids, including blood, plasma, and urine; (iii) samples of tissues that have undergone special interventions, such as drug stimulation, hypoxia, and oxygen-enriched treatment, etc. After having filtered other tissues or diseases out, GSE96804 (24), GSE104948 (13), GSE99339 (25), GSE30528 (9), GSE21785 (26), and GSE47183 (15, 27) were selected for subsequent analysis. As there are fewer control samples in these datasets, we manually retrieved human glomerular datasets as control. Datasets GSE20602 (28), GSE121233 (29), GSE108109 (13), GSE104066 (30), and GSE32591 (31) were included. These dataset have similar characteristics as the control in the DN datasets, which were marked as “glomeruli from living human donor kidney biopsy” and “glomeruli from the unaffected portion of tumor nephrectomies.” Based on all the above datasets, a total of 90 DN and 95 healthy control glomerular samples were included in this study (see Table 1 for details). The samples were collected from multiple platforms, including Affymetrix GeneChip, Human Genome HG-U133A Custom CDF, and the Affymetrix Human Gene 2.1 ST Array.

**Table 1:** The microarray datasets collected and used in this study.

**Dataset ID**	**Experiment platform**	**Disease samples**		**Control samples**		**Package for preprocessing**
		**Type**	**Number**	**Type**	**Number**	
GSE96804 (12)	GPL17586	DN	41	Glomeruli from the unaffected portion of tumor nephrectomies	20	Oligo
GSE104948 (13)	GPL22945	DN	7	Glomeruli from living human donor kidney biopsy	18	Affy
	GPL24120	DN	5	Glomeruli from living human donor kidney biopsy	3	Affy
GSE30122 (9)	GPL571	DN	9	Glomeruli from living human donor kidney biopsy	13	Affy
GSE99339 (25)	GPL19109	DN	7	–	–	Affy
	GPL19184	DN	7	–	–	Affy
GSE47183 (15)	GPL11670	DN	7	–	–	Affy
	GPL14663	DN	7	–	–	Affy
GSE21785 (26)	GPL96	–	–	Glomeruli from living human donor kidney biopsy	6	Affy
GSE20602 (28)	GPL96	–	–	Glomeruli from the unaffected portion of tumor nephrectomies	4	Affy
GSE121233 (29)	GPL17586	–	–	Glomeruli from the unaffected portion of tumor nephrectomies	5	Oligo
GSE108109 (13)	GPL19983	–	–	Glomeruli from living human donor kidney biopsy	6	Oligo
GSE104066 (30)	GPL19983	–	–	Glomeruli from living human donor kidney biopsy	6	Oligo
GSE32591 (31)	GPL14663	–	–	Glomeruli from living human donor kidney biopsy	14	Affy

–* indicates there are no such data*.

### Data Preprocessing and Identification of DEGs

For the preprocessing of a large number of multi-platform microarray datasets, we first used Affy1.64.0 (http://bioconductor.org/packages/release/bioc/html/affy.html) (32) and Oligo 1.50.0 (http://bioconductor.org/packages/release/bioc/html/oligo.html) (33) from Bioconductor in R (3.60) to extract gene expression value. Briefly, after downloading all raw data (Table 1) from the GEO repository, probe expression values were extracted by Affy according to the user guide. After reading the raw data, background correction (rma), normalization (quantiles), probe specific background correction (pmonly), and summary (liwong) were performed to obtain the probe expression value. If the raw data could not be extracted by Affy, the Oligo package was used following the user guide. After reading the raw data, further background subtraction, normalization, and summarization was performed by using rma. All probes were further annotated to genes by their own annotation data. The median of the probe expressions was calculated as the gene expression value. After merging all transcriptomics data into a large sample and removing none of the express genes, Shambhala (https://github.com/oncobox-admin/harmony) was used to perform cross-platform normalization according to the guidance of literature. In this research, a longer P0 was used in Shambhala which was kindly provided by developers and can be found in Supplementary Table 1. This auxiliary calibration dataset contains 13,645 genes which is much longer than what is provided on the website. The levels of each gene expression difference between control and DN were compared by Student's *t*-test coupled with a false discovery rate (FDR) correction. In this study, genes conforming to the fold change (FC) ≥1.5 and *P* < 0.05 (Student's *t*-test adjusted by FDR) were considered as DEGs.

### GO Terms and KEGG Pathway Enrichment of DEGs

GO and KEGG enrichment of the candidate genes were performed using the DAVID online tool (https://david.ncifcrf.gov) (34). GO analysis is a bioinformatics tool that presents information on the biological domain with respect to molecular function (MF), cellular components (CC), and biological processes (BP) (35). KEGG is a database that displays information of system integration gene functions (36). The enrichment significance threshold was set to *P* < 0.05. The visualization of GO enrichment results was conducted by using the GO plot package in the R software (37). To determine the changed tendency of pathways in DN, the Z-score was calculated in each term using the following formula:

$$\begin{array}{l} z-score=\frac{N_{up}-N_{down}}{\sqrt{count}} \end{array}$$

The N_up_ and N_down_ separately represent the number of upregulated and downregulated genes between DN and normal controls, and the count is the number of DEGs belonging to this term (37).

### PPI Network Construction and Module Analysis

Cytoscape 3.8.0 was used for visualization and analysis of the complex network (38). In order to avoid the loss of the protein-protein interaction in a single database, we integrated PPI information collected from multiple databases. We imported network of DEGs by querying the Proteomics Standard Initiative Common QUery Interface (PSICQUIC) which is integrated in Cytoscape (39, 40). Four protein interaction databases were selected for analysis: (i) STRING (https://string-db.org/) (41) which integrates data from high-throughput experiments, text mining, bioinformatics prediction, and interaction databases, (ii) MINT (https://mint.bio.uniroma2.it/) (42) in which PPIs have been confirmed experimentally, (iii) IntAct (https://www.ebi.ac.uk/intact/) (43) which is directly submitted by users, and (iv) Reactome (http://www.reactome.org) (44) which is a pathway database that provides intuitive bioinformatics tools for the visualization, interpretation, and analysis of pathway knowledge. The former three databases focus on exploring the physical interactions between proteins and the last one focuses on biological pathways. After excluding non-human gene information, the analysis results were merged to obtain more comprehensive protein-protein interaction information. In a PPI network, degree and betweenness centrality (BC) are commonly used to evaluate the critical degree of nodes. Degree is the basic index of a node, which is used to indicate the number of links that interact with the node and the network (45). BC measures the importance of nodes based on the shortest paths, which represent the shortest distance between two nodes. A node with a greater BC value has a higher frequency of information exchange within the node (46, 47). In this study, nodes with a high degree and high BC were regarded as key nodes, and genes whose BC and degree were both in the top 10% in the total nodes of the network were regarded as important genes. In this study, we computed the properties of nodes and measured the default parameters with Cytoscape. Next, we used the Cytoscape plug-in Molecular Complex Detection tool (MCODE; version 1.5.1) (48) to identify the most important module in the network map. The criteria for MCODE analysis were a degree of cut-off = 2, MCODE scores >6, maximum depth = 100, node score cut-off = 0.2, and k-score = 2(48).

### Core Gene Identification

We selected DEGs that met the following three constraints as core genes: (i) DEGs that had a large fold change (top 100); (ii) the gene was located in key module; and (iii) nodes with top 10% BC values and degree determined by Cytoscape software.

### Immune Cells Infiltration in DN Glomerular Tissue

**Figure 1:**
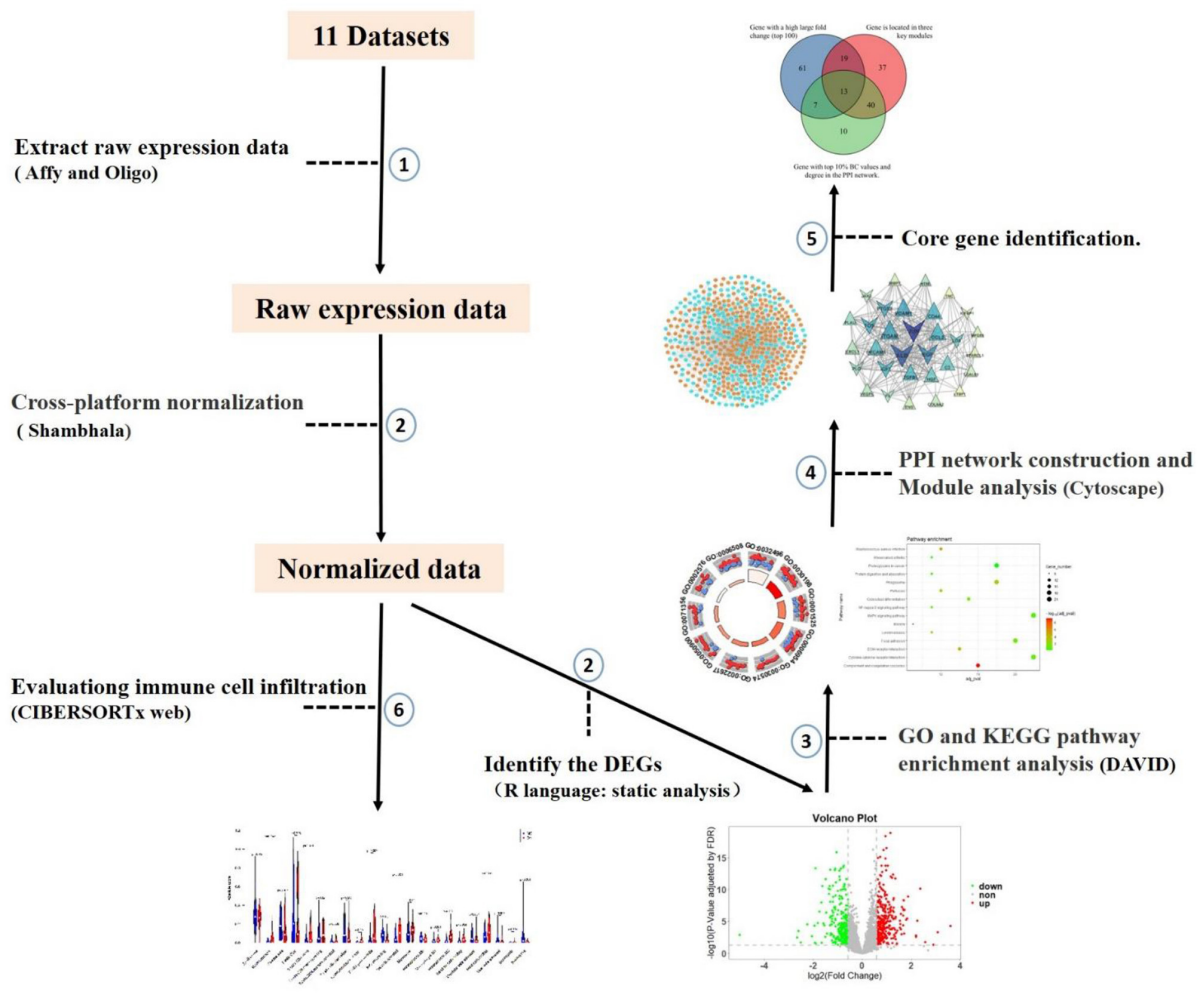
The workflow of microarray data preprocessing and subsequent analysis in this study. We selected 11 datasets based on the constraints. Firstly, raw expression data were extracted after background correction and quality control. Processing was carried out using the R package, and then the data were cross-platform normalized by coordination and transformation using Shambhala. Then DEGs of DN glomerular tissue and healthy control tissue were identified by static analysis. Enrichment results were obtained using DAVID, then PPI networks and modules were constructed, and core genes were identified. Finally, the dataset was brought into the CIBERSORTx web portal to evaluate immune cell infiltration.

CIBERSORTx (https://cibersortx.stanford.edu) (49) is a digital cytometry program that uses a machine learning method. It can provide an estimation of the abundances of member cell types in a mixed cell population by using gene expression data. We used a validated leukocyte gene signature matrix that contained 547 genes to distinguish 22 human hematopoietic cell phenotypes to identify glomerular immune cells infiltration. Seven T cell types, naive and memory B cells, plasma cells, natural killer (NK) cells, and myeloid subsets infiltration alteration were identified (50). After uploading the cross-platform normalized data to CIBERSORTx, permutations were set at 100 and absolute mode was selected. Absolute mode scales relative cellular fractions into a score that reflects the absolute proportion of each cell type in a mixture. When the *p* < 0.05, it indicates that the infiltration rate of the 22 immune cells types analyzed by CIBERSORTx is accurate. The accurately identified immune cell infiltration was further compared between normal control and DN by Wilcoxon signed-rank test. The steps of the whole process are shown in Figure 1.

## Results

### Identification of DEGs

**Figure 2:**
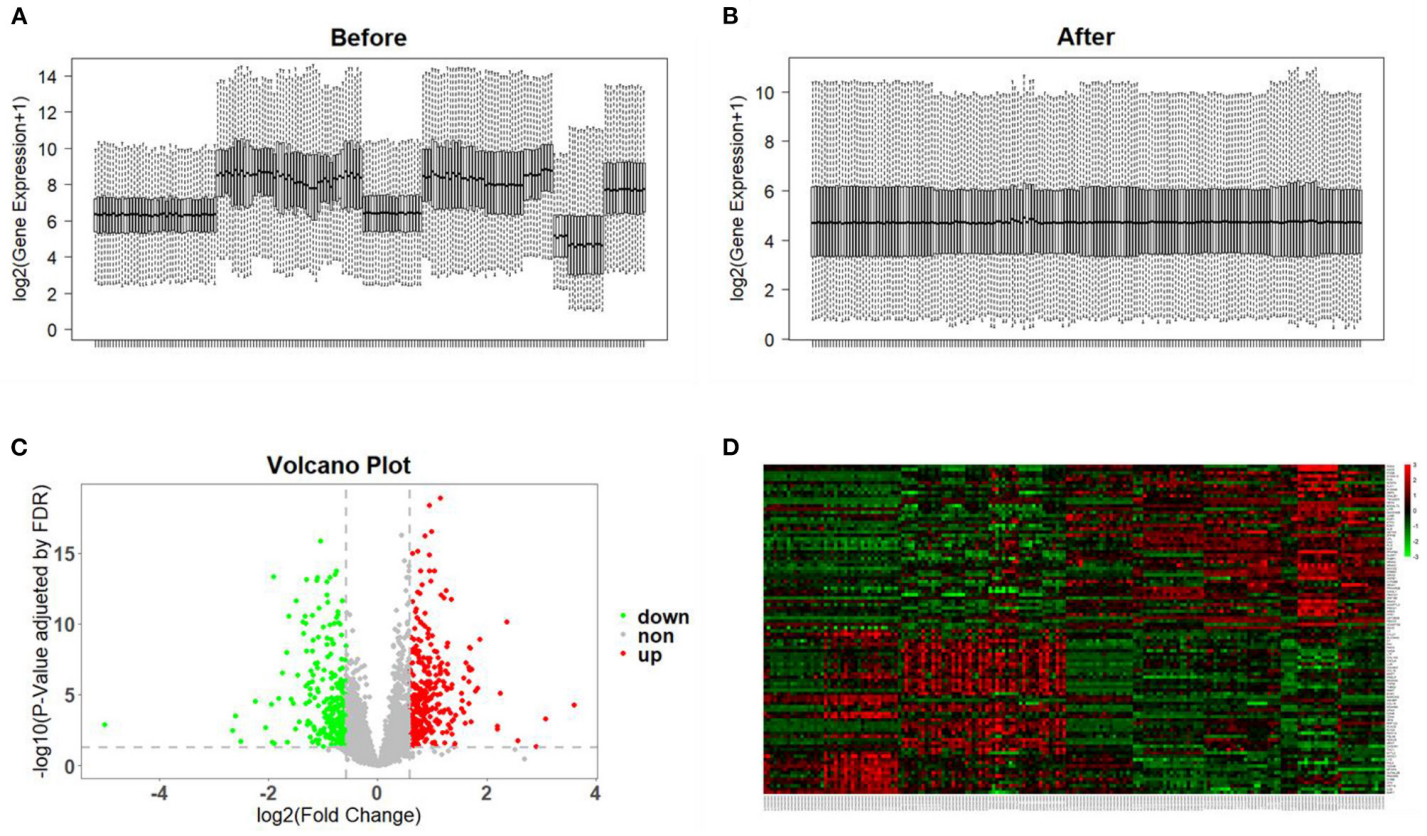
Data preprocessing and identification of DEGs. **(A,B)** Gene expression data before and after normalization. The horizontal axis represents the sample symbol and the vertical axis represents the gene expression values. The black line in the box plot represents the median value of gene expression. **(C)** Volcano plot analysis of DEGs. Red represents high expression, green represents low expression, and gray represents no difference. **(D)** The heatmap of top 100-fold-change DEGs. Red areas represent highly expressed genes and green areas represent lowly expressed genes in glomerular tissues from DN patients compared with normal controls.

All microarray datasets were standardized, and the results before and after standardization are shown in Figures 2A,B. According to values of *p* < 0.05 and FC ≥ 1.5, a total of 578 genes were identified to be differentially expressed in the DN group, including 334 upregulated and 244 downregulated genes (Figure 2C and Supplementary Table 2). DEGs with the top 100-fold change are shown in the heatmap (Figure 2D).

### GO and KEGG Pathway Analysis

**Figure 3:**
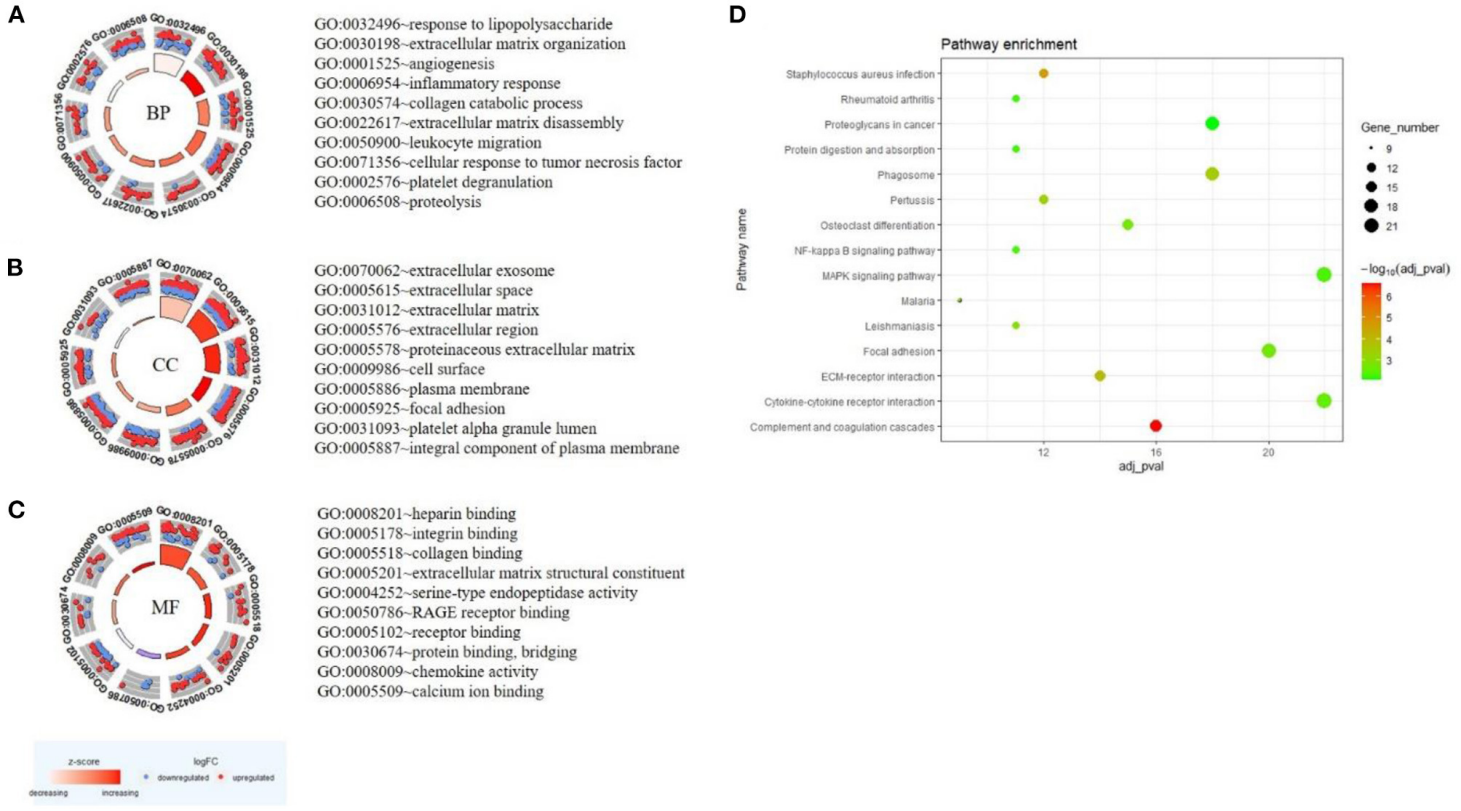
GO and KEGG enrichment result of DEGs. The result of the top 10 enrichment genes is shown in the GOCircle plot: BP **(A)**, CC **(B)**, MF **(C)**. The inner ring is a bar plot where the bar height indicates the significance of the term (*p*-value) and the color indicates the z-score. The outer ring displays scatterplots of the expression levels (logFC) for the genes in each term. The blue node is the downregulated gene, and the red node is the upregulated gene. **(D)** The top 15 KEGG enrichment results. The x-axis represents gene number and the y-axis represents KEGG terms. The size of the circle represents gene count. Circles of different colors represent different adjusted *p*-values.

GO analysis was performed based on the 578 DEGs, and circle graphs show the top 10 entries of each term. BP demonstrated that the DEGs were enriched in lipopolysaccharide, extracellular matrix organization, angiogenesis, inflammatory response, leukocyte migration, and platelet degranulation, etc. (Figure 3A). Variations in DEGs linked with CC were extracellular exosome, extracellular matrix, focal adhesion, and platelet alpha granule lumen, etc. (Figure 3B). Regarding MF, DEGs were significantly enriched in heparin binding, integrin binding, collagen binding, and extracellular matrix structural constituent, etc. (Figure 3C). Analysis of KEGG pathways indicated that canonical pathways associated with DEGs were complement and coagulation cascades, staphylococcus aureus infection, and ECM-receptor interaction, etc. The top 15 KEGG enrichment results are shown in Figure 3D. The complete enrichment analysis results are in Supplementary Table 3.

### PPI Network Construction and Module Screening

**Figure 4:**
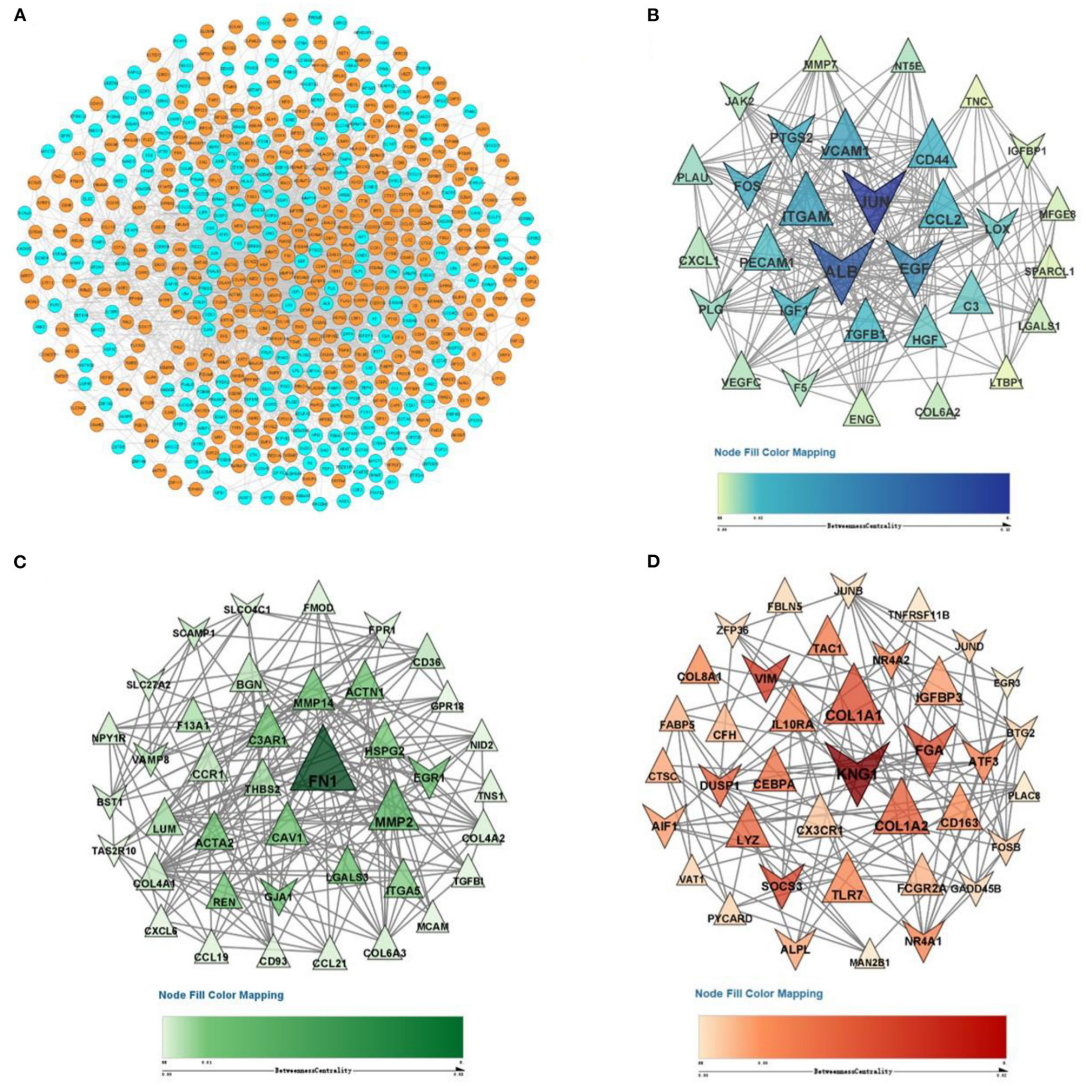
PPI network and three significant modules of DEGs. **(A)** PPI network of DEGs created by merging the STRING, MINT, IntAct, and Reactome databases. The orange purple nodes represent upregulated DEGs, and blue nodes represent downregulated DEGs. A total of 578 DEGs formed a PPI network consisting of 557 nodes and 3,882 edges. **(B)** The most significant module identified by MCODE (score = 16.867). **(C)** The second most significant module identified by MCODE (score = 10.051). **(D)** The third most significant module identified by MCODE (score = 6.216). The size of the nodes corresponds to their degree.

The DEG expression products in DN were constructed into PPI networks by merging the STRING, MINT, IntAct, and Reactome databases in Cytoscape software. By removing the separated and separately connected nodes, a complex network of DEGs was constructed and is presented in Figure 4A. Three modules were identified by MCODE (Figures 4B–D). Module 1 (score = 16.867) was composed of 31 nodes and 253 edges, module 2 (score = 10.051) was composed of 40 nodes and 196 edges, and module 3 (score = 6.216) was composed of 38 nodes and 115 edges.

### Core Gene Identification

**Figure 5:**
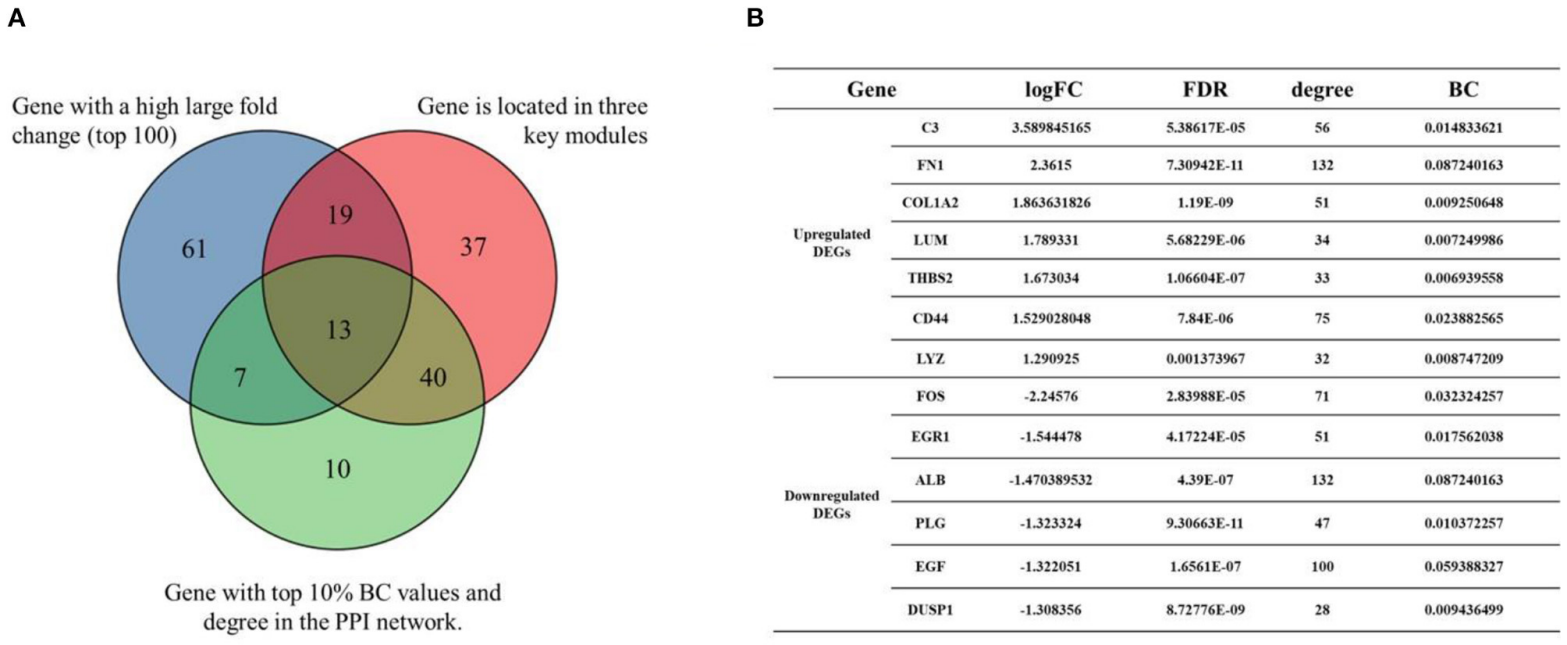
Venn diagram of core genes. **(A)** The blue circle represents DEGs that had a high large fold change (top 100). The red circle represents DEGs that were located in the three key modules. The green circle represents DEGs that had the top 10% BC values and degree in the PPI network. **(B)** Details of core genes (FC, FDR adjusted *p*-value, node degree, node BC value).

In this study, *C3, CCL21, SLC34A2, C7, ALB, ESM1, ATF3, EGR1* etc. had large fold change, 109 genes (*JUN, ALB, EGF, VCAM1, ITGAM*, etc.) were located in the three key modules, 70 genes (*JUN, ALB, FN1, EGF, VCAM1, ITGAM, FOS*, etc.) were nodes in the PPI network with the top 10% of BC value and degree. The Venn diagram presented illustrates the overlaps between DEGs (Figure 5A). As shown in the Venn diagram, we selected 13 eligible DEGs as core genes, including complement C3 (*C3*), fibronectin 1 (*FN1*), collagen type I alpha 2 chain (*COL1A2*), lumican (*LUM*), thrombospondin 2 (*THBS2*), CD44 molecule (*CD44*), lysozyme (*LYZ*), Fos proto-oncogene (*FOS*), early growth response 1 (*EGR1*), albumin (*ALB*), plasminogen (*PLG*), epidermal growth factor (*EGF*), and dual-specificity protein phosphatase-1 (*DUSP1*). The details of core genes are shown in Figure 5B.

### Infiltration of Immune Cells in DN

**Figure 6:**
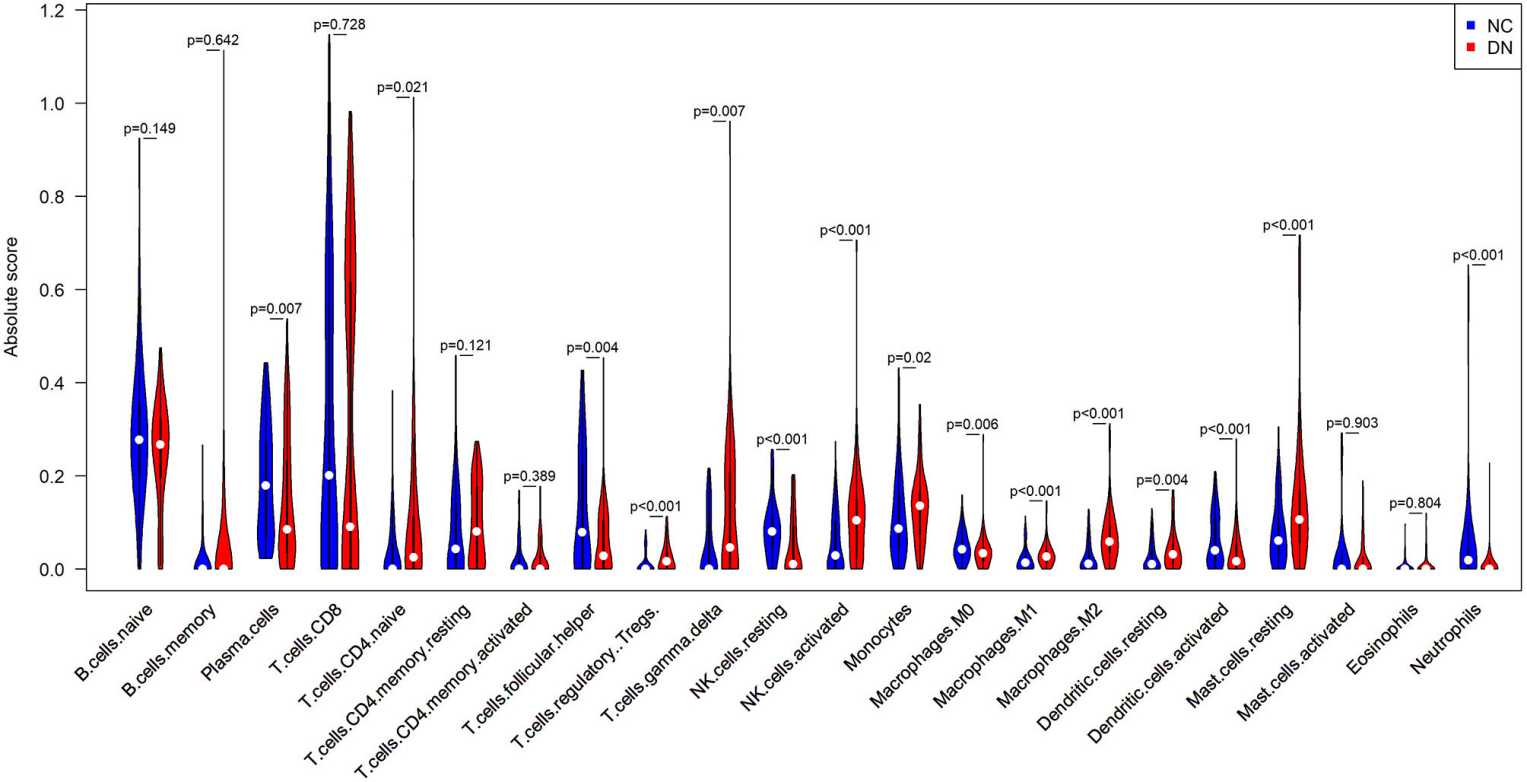
Comparison of infiltrated immune cell subpopulations in glomerulus tissues with or without DN. Violin plot of immune-infiltrating lymphocytes between DN glomerular tissues and healthy control glomerular samples, in which the red represents DN samples, and the blue represents control samples.

Since inflammation is enriched in GO and KEGG, it will be interesting to specify which immune cells infiltrated the glomerular under DN. CIBERSORTx is a bioinformatics tool that can specifically analyze the infiltration of immune cells in tissues. The results of CIBERSORTx analysis showed that there were 172 samples of glomerulus transcriptomic data at *p* < 0.05 (86 control and 86 DN). Indicating that most of the infiltration rates of the 22 immune cells types analyzed by CIBERSORTx were accurate. Compared with normal control glomerular tissues, the infiltration of plasma cells, follicular helper T cells, resting NK cells, macrophages M0, activated dendritic cells (DCs), and neutrophils were reduced in glomerular tissues affected by DN. Infantile CD4+ T cells, regulatory T cells, γδT cells, activated NK cells, macrophages M1, macrophages M2, resting DCs, and mast cells were increased in DN glomerular tissues (Figure 6).

## Discussion

DN is a serious complication of long-term diabetes mellitus (DM) and a growing global economic burden (51). The glomerulus plays a key role in the development of DN. However, due to the complexity of etiology and ethnic differences, our understanding of the molecular mechanism in DN glomerular tissue is still incomplete. Therefore, it is urgent to explore the new molecular mechanism which may help DN treatment and diagnosis.

In this study, we used bioinformatics methods to analyze GEO transcriptomics datasets before December 2020, and attempted to explore the potential molecular mechanisms of the DN glomerulus. A total of 578 DEGs were identified in the glomerular samples between DN and normal samples, including 334 upregulated and 244 downregulated genes. Thirteen core genes were finally identified, including *C3, FN1, COL1A2, LUM, THBS2, CD44, LYZ, FOS, EGR1, ALB, PLG, EGF*, and *DUSP1*.

Among these core genes, some have been shown to play an important role in the pathogenesis of DN, C3 (52, 53), ALB (54–56), EGF (57), EGR1 (58–61), COL1A2 (62), FN1 (63, 64), CD44 (65, 66), FOS (67), PLG (68, 69), and DUSP1 (70). It is well-known that inflammation and fibrosis play an important role in the pathogenesis of the DN glomerulus (71).

Among these 13 core genes, there is little known about what roles *LYZ, LUM*, and *THBS2* play in the development of DN. LYZ, which encodes lysozymes, is an antimicrobial agent found in human milk. It is also found in the spleen, lungs, kidneys, white blood cells, plasma, saliva, and tears. Gallo et al. found that LYZ downregulated the production and release of inflammatory mediators [such as interleukin (IL)-6] induced by late glycosylation end products in *in vitro* models of human proximal renal tubular epithelial cells, and prevented the recruitment of some macrophages at the inflammatory site (72). Indicating that locally expressed LYZ may take part in the pathogenesis of DN. LUM encodes members of the leucine-rich small proteoglycan (SLRP) family, which includes decorin, biglycan, and fibromodulin, etc. (73). The protein expressed by this gene partially binds collagen fibers, and highly charged hydrophilic glycosaminoglycans regulate the spacing between fibers (74). It has been reported that the LUM protein and its family member decorin accumulate strongly in the advanced glomerulosclerosis stage of DN (75). Decorin greatly affects the progression of DN by forming the ternary complex of decorin-type I collagen-transforming growth factor, beta (TGF-β) (75). It is speculated that LUM may also promote the development of DN by interacting with TGF-β. THBS2 proteins belong to the thrombospondin family. As a relatively special member of this family, it has an anti-angiogenic effect and interacts with various cell receptors and growth factors to regulate cell proliferation, apoptosis, and adhesion (76). It has been shown that THBS2 plays an important role in acute kidney injury (AKI) (77). It also has been found differently expressed in the plasma of type 2 diabetes patients (78). This indicates that THBS2 plays an important role in DN.

We enriched the GO terms and KEGG pathway of DEGs. Among the enriched pathways, inflammatory response, leukocyte migration, platelet degranulation, and platelet alpha particles attracted our attention. We further used CIBERSORTx to identify immune cell infiltration in DN glomerular tissues. Three types of T cells increased infiltration in the DN tissues, including naive CD4+ T cells, regulatory T cells, and γδT cells. The originally resting NK cells in the tissues were activated, and the macrophages were also differentiated from resting M0 into the classically activated and pro-inflammatory M1 and the alternatively activated M2. The active DCs were reduced to resting. The infiltration of mast cells was increased and the infiltration of plasma cells and neutrophils was decreased in DN glomerular tissue. These results imply that humoral immunity and cellular immunity is altered in DN patient glomerular tissue. These results imply that there may be crosstalk among the glomerulus, platelets, and immune cells.

Glomerular cells can recruit immune cells and activate platelets in DN. Glomerular cells suffer from oxidative stress (79), advanced glycation end products (AGEs) (80), abnormal lipid metabolism (81), and other damages in DN. These will lead the glomerular cells to lose their function and induce fibrosis (68). Injured glomerular mesangial cells, glomerular endothelial cells, and podocytes can produce inflammatory and adherence factors to recruit and activate immune cells (79, 82). These factors such as C-C motif chemokine ligand 2 (CCL2) (83–86), C-X3-C motif chemokine receptor 1 (CX3CR1) (83), inter-cellular adhesion molecule-1 (ICAM-1) (87, 88), vascular cell adhesion molecule-1 (VCAM-1) (89), and tumor necrosis factor-alpha (TNF-α) (90, 91) will recruit and activate lymphocytes, monocytes, and other immune cells (83). Injured glomerular mesangial cells, glomerular endothelial cells, and podocytes can also activate platelets (92, 93). In the development of DN, collagen is accumulated in the glomerulus (94, 95). Collagen has long been considered as an important activator of platelet activation. Collagen can directly bind to the glycoprotein VI (GPVI) receptor or integrate the von Willebrand factor (vWF) to activate the glycoprotein Ib-IX-V complex (GPIb-IX) receptor to activate platelets (96, 97). In DN, the increase of AGEs (98), chemokines (such as CCL2, C-X-C motif chemokine ligand 1 (CXCL1)) (99–101), very low density lipoprotein (VLDL) (102), and abnormal metabolism of nitric oxide (NO) can also active platelets (92, 103–105).

Recruited immune cells can release a variety of chemokines, these will damage glomerular cells, cause fibrosis in DN, and activate platelets. Recruited macrophages can be induced by locally secreted TNF-α, and differentiated from resting M0 to activated pro-inflammatory M1 and M2 (90, 91). Once activated, macrophages will release reactive oxygen species (ROS), IL-1, TNF-α, complement factors, and metalloproteinases (106). Recruited mast cells and macrophages can release CXCL1 (107–109). Recruited CD4+ T cells (110), γδ T cells, and NK cells (111) secrete inflammatory factors (interferon gamma (IFN-γ) and IL-17A) (112, 113) and chemokines to promote the proliferation and differentiation of B cells and the formation of immune complexes (114). We all know those factors will damage the glomerulus, cause fibrosis, and promote the progression of DN (82, 83). Immune cells release chemokines (such as CCL2, CXCL1), and ROS can also activate platelets (99–101, 115).

Activated platelets may further activate other platelets (94), recruit immune cells, damage glomerular cell, and cause fibrosis in DN. Once platelets are activated, platelets will express the CD36 molecule (CD36), protein kinase C eta (PRKCH), and coagulation factor II thrombin receptor like 2 (F2RL2) on the surface of platelets, which will cause more platelets to be activated (116–123). Platelet hyper function is observed in DM (92, 93) and DN (93) patients, indicating that platelets may play an important role in the development of DN. Activated platelets release a variety of pro-inflammatory cytokines (TNF-α, P-selectin, TGF-β, FN1, ILs, VCAM-1) and chemokines (CCL-2, CXCL1, and CX3CR1) (96). As described before, some of those factors can recruit and active immune cells (124), and those active immune cells will damage glomerular cells. Some of these factors even can damage glomerular cells directly, such as TGF-β (125), TNF-α (126), ILs (127), and FN1 (63).

**Figure 7:**
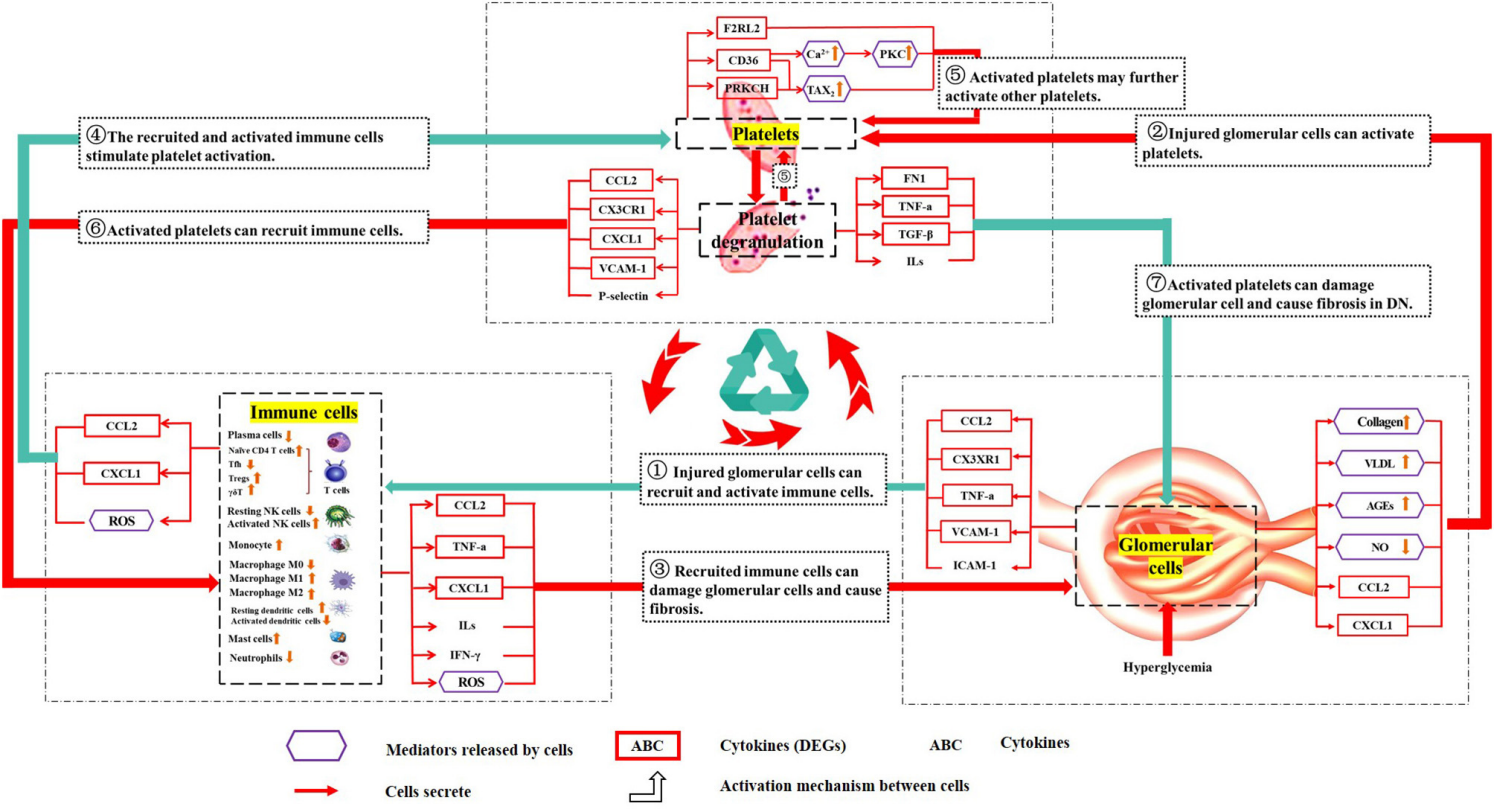
Schematic diagram describing the crosstalk among platelets, immune cells, and the glomerulus. The purple hexagonal box lines represent cellular mediators. The red box indicates the protein expressed by DEGs. The frameless protein was not expressed by DEGs. “ → ” cells that secrete; “
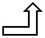
” the activation mechanism between cells (the red line is glomerular cells-immune cells-platelets, and the blue line is glomerular cells-platelets-immune cells). Injured glomerular cells can recruit and activate immune cells. ① Injured glomerular cells can recruit and activate immune cells. ② Injured glomerular cells can activate platelets. ③ Recruited immune cells can damage glomerular cells and cause fibrosis. ④ The recruited and activated immune cells stimulate platelet activation. ⑤ Activated platelets may further activate other platelets. ⑥ Activated platelets can recruit immune cells. ⑦ Activated platelets can damage glomerular cells and cause fibrosis in DN. The above is the hypothetical crosstalk among the glomerulus, immune cells, and platelets.

Therefore, there may be positive feedback among the glomerulus, platelets, and immune cells. This vicious cycle may damage the glomerulus for a long time even after the initial high glucose damages have been removed. This may be a reason why renal damage in DN patients still progresses even after blood glucose was strictly controlled. The hypothesized crosstalk among platelets, immune cells, and glomerular cells are shown in the schema (Figure 7).

## Conclusion

In summary, we found three core genes that may be associated with the pathogenesis of DN (*LYZ, LUM*, and *THBS2*). Furthermore, our further bioinformatics analysis suggested that there might be positive feedback among platelets, immune cells, and the glomerulus. And this feedback may damage the glomerulus for a long time even after the initial high glucose damages have been removed. These findings may provide new ideas for the pathogenesis and treatment of DN. However, due to the lack of experimental verification in this study, further studies are needed.

## Data Availability Statement

The related microarray datasets GSE96804, GSE104948, GSE99339, GSE30528, GSE21785, GSE47183, GSE20602, GSE121233, GSE108109, GSE104066 and GSE32591 were downloaded from the GEO (https://www.ncbi.nlm.nih.gov/gds/).

## Author Contributions

ZL, HS, and HZ conceived and designed the study. XY, HS, FC, HH, BL, XZ, HZ, and ZL performed bioinformatics and correlation analyses. XY wrote the first draft. ZL and HS revised the manuscript. All authors contributed to the article and approved the submitted version.

## Conflict of Interest

The authors declare that the research was conducted in the absence of any commercial or financial relationships that could be construed as a potential conflict of interest.

## Supplementary Material

Supplementary Table 1 Long P0 datasets.

Supplementary Table 2 Identification of DEGs.

Supplementary Table 3 The complete enrichment analysis results.
